# Does COVID-19 infection change the need for future surgical interventions? An exploratory analysis.

**DOI:** 10.12688/f1000research.74861.1

**Published:** 2021-11-17

**Authors:** Blayne Welk, Lucie Richard

**Affiliations:** 1 Surgery, Western University, London, ON, Canada; 2 ICES, London, Canada

**Keywords:** Surgery, SARS-CoV-2, Covid-19, Renal stone disease

## Abstract

**Background: **It is unknown whether recovery from coronavirus disease 2019 (COVID-19) infection leads to an increased need for common surgical procedures. Our objective was to conduct an exploratory analysis of surgical procedures performed after a documented COVID-19 infection.

**Methods:** We conducted a retrospective cohort study using routinely collected data from the province of Ontario, Canada. We identified individuals with a positive COVID-19 test between February 1 2020 and May 31 2020, and matched them 1:2 with individuals who had a negative COVID-19 test during the same period. We used physician billing codes to identify the ten most frequent surgical procedures in the COVID-19 cohort. An at-risk period 30 days after the first positive COVID-19 swab (or matched index date in the control group) until November 30 2020 was used. Cox proportional hazard models (adjusted for important baseline differences) are reported with hazard ratios (HR) and 95% confidence intervals.

**Results: **After exclusions and matching, we had 19,143 people in the COVID-19 cohort, and 38,286 people in the control cohort. The top ten surgical procedures were hand/wrist fracture fixation, cesarean-section, ureteral stent placement, cholecystectomy, treatment of an upper tract urinary stone, hysterectomy, femur fracture repair, hip replacement, transurethral prostatectomy, and appendectomy. There was a significantly reduced hazard of requiring upper tract renal stone surgery (adjusted hazard ratio [aHR] 0.50, 95% confidence interval [CI] 0.29-0.87) or ureteral stent placement (aHR 0.54, 95%CI 0.36-0.82), or undergoing a cholecystectomy (aHR 0.43, 95%CI 0.26-0.71) among those with a prior positive COVID-19 test.

**Conclusions: **After a COVID-19 infection there is not an increased risk of needing several common surgical procedures. There appears to be a reduced risk of renal stone disease treatment and ureteral stent placement, and a reduced risk of cholecystectomy, however understanding the reasons for this will require further study.

## Introduction

The coronavirus disease 2019 (COVID-19) global pandemic has led to fundamental shifts in everyday life around the world. Immense amounts of clinical research have examined the potential mechanisms and sequela of COVID-19 complications. ^1^ This includes a significant volume of research on acute, and long-term complications of COVID-19. As a previously unknown disease, an important part of this research process is exploratory analyses to determine which health conditions may be more prominent after a COVID-19 infection; this led to important studies which have identified the risk of long-term sequela such as neurologic, metabolic, cardiovascular and gastrointestinal disorders that are now recognized as being part of long-COVID-19. ^2^^,^^3^

For surgeons, most of the early study of COVID-19 centered around how surgical practices changed in response to the pandemic, ^4^ safe operating room practices, ^5^ or the abnormally high surgical mortality rate associated with operating on a COVID-19 patient. ^6^ However, little research has examined if COVID-19 puts patients at increased risk of requiring specific surgical procedures after the period of acute infection. Given the wide-ranging impact of COVID-19 across several different organs, and the fact there is still much to be understood about long COVID-19, it is possible that patients with a previous COVID-19 infection are at an elevated risk of diseases that are managed operatively. Knowledge of this would be important for patients, physicians treating COVID-19 patients, and the surgical community.

Our objective was to use routinely collected administrative data to conduct an exploratory analysis of surgical procedures performed after a documented COVID-19 infection.

## Methods

This is a retrospective cohort study. We made use of routinely collected data from the province of Ontario, Canada. All the datasets were linked using an encoded version of a unique patient identifier. All data was analysed at ICES Western. Ontario (Canada’s largest province with a population of 14.7 million people in 2020) has a single universal healthcare system, meaning this data is population based. The use of the data in this study was authorized under Ontario’s Personal Health Information Protection Act (Section 45), which does not require approval by a Research Ethics Board, or patient consent. ^7^ A completed STROBE checklist, data collection plan and example analytic code for this study can be found as *Extended data.* ^28^

### Datasets

We made use of several different datasets for this study. First, we used the Ontario Laboratories Information System (OLIS) to determine COVID-19 polymerase chain reaction (PCR) results. ^8^ This dataset contains over 97% of the provincial COVID-19 PCR tests. Second, we used the Ontario Health Insurance Plan (OHIP) records, which contains information for all services provided through the province’s publicly funded health insurance system. Physicians must submit billing codes to this database to be compensated for medical services they provide; these OHIP fee codes have a high accuracy. ^9^ Third, the Discharge Abstract Database (DAD)/Same Day Surgery (SDS)/National Ambulatory Care Reporting System (NACRS) datasets are compiled by the Canadian Institute for Health Information. They receive administrative, clinical (diagnoses and procedures/interventions), and demographic information for patients who have a hospital admission, same day surgery, or emergency room visit (respectively). Re-abstraction studies have demonstrated that diagnostic codes (89% exact match, 95% ICD10 code group match) are quite accurate. ^10^ Finally, the Registered Persons Database (RPDB) provides basic demographic information (such as age and sex) for all people who are permanent citizens of Ontario.

### Study population

We first identified all people in Ontario who had a positive COVID-19 nasopharyngeal PCR test between February 1 2020-May 31 2020 (COVID-19 cohort) using OLIS. We used the date of the positive nasopharyngeal swab as the index date of the infection. Using OLIS and RPDB, we excluded patients who were less than 18 years of age, those that had evidence of a previous positive COVID-19 PCR test in the 3 months prior, and those that died within 30 days of the COVID-19 diagnosis (as we were only interested in the period after acute COVID-19 infection). We also excluded residents of long-term care, as they were disproportionally affected by COVID-19, ^11^ and in many instances have an intrinsic bias against surgical interventions due to competing medical issues, and advance care directives.

To create a comparison group (control cohort), we started by identifying all people that were alive in Ontario as of February 1, 2020, and had at least one negative COVID-19 nasopharyngeal PCR test between February 1 2020-May 31 2020. We used those with documented negative COVID-19 PCR tests for comparison, as there are systemic differences in the types of people that had access to and underwent COVID-19 testing, and this can introduce collider bias. ^12^ For each of the people who met these inclusion criteria, we used the negative test date as their index date. We excluded people who had evidence of a COVID-19 infection between February 1 2020-May 31 2020, and then used the same exclusion criteria as we did for the COVID-19 cohort (age <18 years, those that died during the first 30 days following the index date and those residing in long-term care).

We matched (without replacement) one person from the COVID-19 cohort to two people from the control cohort based on the index date (±1 week), age (±1 year), and sex. We used two years of data from our datasets prior to the index date and three validated datasets (to identify patients with chronic obstructive pulmonary disease, ^13^ hypertension, ^14^ and diabetes ^15^) to measure several baseline characteristics relevant to COVID-19 infection ^16^ (coding details have been previously described ^17^).

### Outcomes

Our primary outcome was the occurrence of surgical procedures 30 days after the index date. As this was an exploratory analysis, we did not identify specific surgical procedures *a priori*, but instead examined the top ten most frequent surgical procedures that occurred in the COVID-19 cohort during the follow-up period. These procedures were identified using OHIP fee codes (listed in the results table for the ten surgeries of interest). Each patient was observed for surgical procedures from the index date + 30 days, until November 30 2020 (with a follow-up period of six to ten months). Patients were censored if they died, or in the case of the control patients, if they were diagnosed with COVID-19 between May 31 and November 30 2020.

### Statistical analysis

We compared baseline characteristics between the matched COVID-19 and the control cohorts using standardized differences; >10% is a potentially meaningful difference. ^18^ We used PROC PHREG ( SAS 9.4, SAS institute, Cary, NC, USA) to create Cox proportional hazards models with robust variance estimation to account for the correlation introduced with matching. We adjusted statistical models for any differences in the baseline characteristics which had a standardised difference >10%. Results are reported as hazard ratios (HR) and 95% confidence intervals (CI), and we considered two-tailed p-values <0.05 statistically significant.

## Results

**Figure 1.**
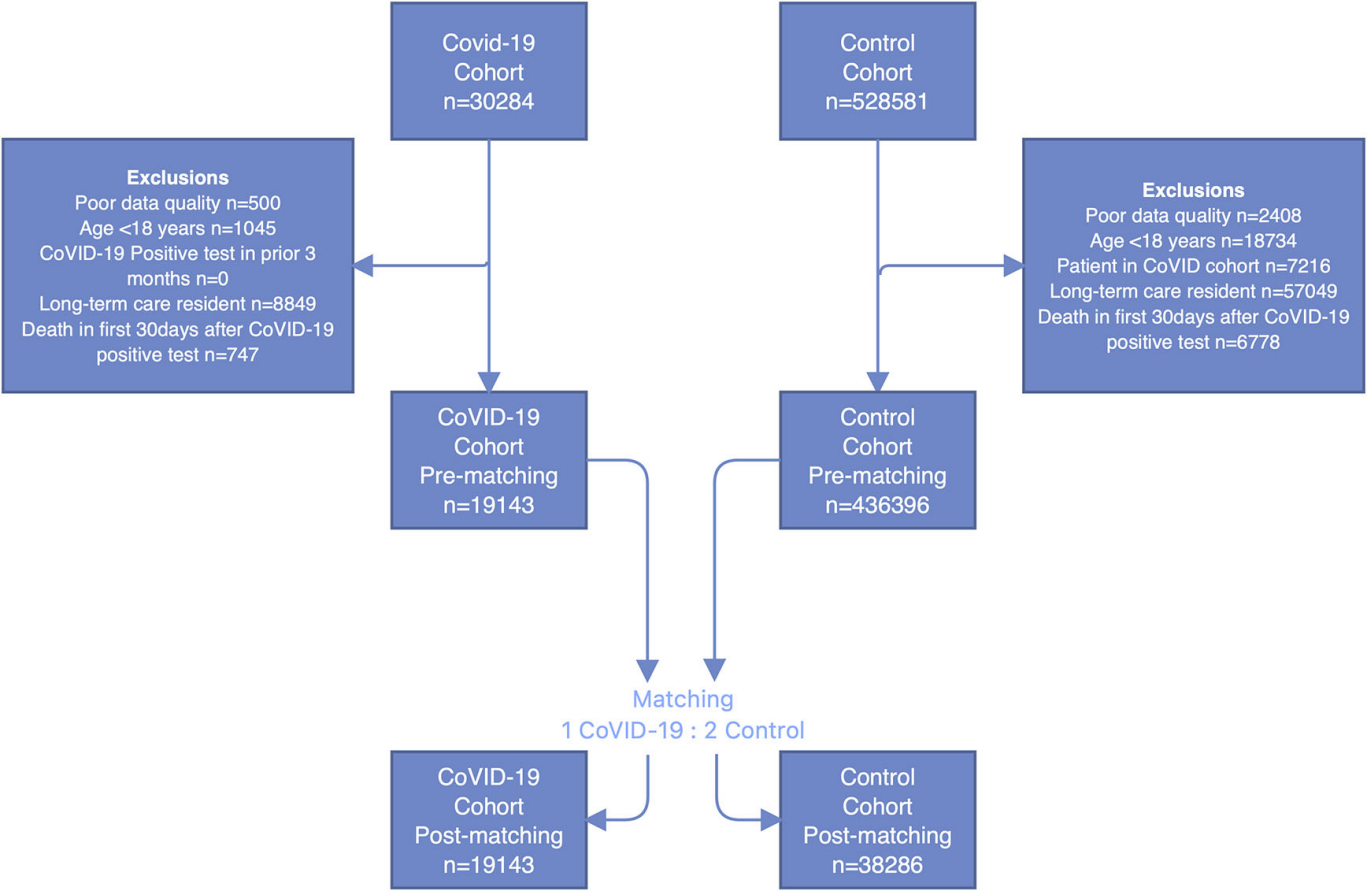
Cohort creation flowchart.

We identified 30,284 people who had a positive COVID-19 test during the four-month study accrual period; of these 19,143 remained after our exclusion criteria. There were 528,581 people who had a negative COVID-19 test during the four-month study accrual period; 436,396 remained after applying the exclusion criteria, and 38,286 were matched to our COVID-19 positive patients (details in Figure 1).

The COVID-19 and control cohorts had similar age ranges and sex composition due to the matching. Other baseline variables were generally similar, other than a higher rate of chronic obstructive lung disease and heart disease among the control cohort ( Table 1). The top ten surgical procedures that were identified were hand/wrist fracture fixation, Cesarean-section, ureteral stent placement, cholecystectomy, treatment of an upper tract urinary stone, hysterectomy, femur fracture repair, hip replacement, transurethral prostatectomy, and appendectomy. After adjusting for COPD cancer, and heart disease, there was a significantly reduced hazard of requiring upper tract renal stone surgery or ureteral stent placement, or undergoing a cholecystectomy among people who had a history of a positive COVID-19 test ( Table 2).

**Table 1.** Description of the matched control and coronavirus disease 2019 (COVID-19) cohorts. SD = standard deviation.

	Control cohort	COVID-19 cohort	Standardised differences
	N = 38,286	N = 19,143	
**Demographics**			
Age at Index Date			
Mean (SD)	48.75 ± 17.56	48.75 ± 17.57	0
<30 yrs	6,432 (16.8%)	3,216 (16.8%)	0
30 to 39 yrs	6,175 (16.1%)	3,087 (16.1%)	0
40 to 49 yrs	7,057 (18.4%)	3,529 (18.4%)	0
50 to 59 yrs	8,614 (22.5%)	4,306 (22.5%)	0
60+ yrs	10,008 (26.1%)	5,005 (26.1%)	0
Female, N (%)	17,234 (45.0%)	8,617 (45.0%)	0
Income quintile, N (%)			
Missing	131 (0.3%)	34 (0.2%)	0.03
Quintile 1 (lowest)	8,677 (22.7%)	5,361 (28.0%)	0.08
Quintile 2	7,940 (20.7%)	4,020 (21.0%)	0.01
Quintile 3	7,566 (19.8%)	3,890 (20.3%)	0.01
Quintile 4	7,228 (18.9%)	3,151 (16.5%)	0.06
Quintile 5 (highest)	6,744 (17.6%)	2,687 (14.0%)	0.1
Month of COVID-19 diagnosis			
Feb	-	13 (0.1%)	-
March	-	2,819 (14.7%)	
April	-	9,191 (48.0%)	
May	-	7,120 (37.2%)	
**Comorbidities in the previous 2 years, N (%)**			
Chronic obstructive pulmonary lung disease	2,009 (5.2%)	422 (2.2%)	0.16
Diabetes	5,036 (13.2%)	2,865 (15.0%)	0.05
Heart Failure	1,437 (3.8%)	458 (2.4%)	0.08
Hypertension	6,448 (16.8%)	3,646 (19.0%)	0.06
Heart Disease	1,469 (3.8%)	419 (2.2%)	0.10
Stroke/Transient ischemic attack	1,020 (2.7%)	406 (2.1%)	0.04
Obesity	1,732 (4.5%)	935 (4.9%)	0.02
Chronic kidney disease	1,347 (3.5%)	451 (2.4%)	0.07
Cancer	2,085 (5.4%)	679 (3.5%)	0.09

**Table 2.** Adjusted hazard ratios (aHR) for the ten most frequent surgical procedures in the coronavirus disease 2019 (COVID-19) cohort compared to the control cohort. aHR = adjusted hazard ratio; CI = confidence interval.

	Control cohort		COVID-19 cohort		aHR (95% CI) Control cohort is the reference group ^a^
	38,286		19,143		
	n	%	n	%	
**Primary exploratory analysis**					
Hand/Wrist fracture repair	105	0.27%	47	0.25%	0.976 (0.687-1.385)
Cesarean-section	68	0.18%	41	0.21%	1.130 (0.766-1.666)
Ureteral stent placement	106	0.28%	30	0.16%	0.543 (0.358-0.824) *
Cholecystectomy	90	0.24%	21	0.11%	0.431 (0.262-0.708) *
Treatment of an upper tract urinary stone	60	0.16%	16	0.08%	0.503 (0.289-0.873) *
Hysterectomy	35	0.09%	16	0.08%	0.899 (0.503-1.606)
Femur fracture repair	21	0.05%	10	0.05%	1.058 (0.486-2.301)
Hip replacement	26	0.07%	10	0.05%	0.801 (0.386-1.662)
Transurethral prostatectomy	15	0.04%	10	0.05%	1.718 (0.734-4.021)
Appendectomy	37	0.10%	10	0.05%	0.513 (0.253-1.041)
**Post-hoc analysis**					
Nephrostomy tube insertion 30 days after index date until the end of the follow-up period	26	0.07%	N<6 ^b^	<0.03%	0.251 (0.075-0.840) *
Early Ureteral stent placement (between the index date and index date + 30 days)	49	0.13%	N<6 ^b^	<0.03%	0.069 (0.020-0.290) *
Early Treatment of an upper tract urinary stone (between the index date and index date + 30 days)	35	0.09%	N<6 ^b^	<0.03%	0.102 (0.020-0.430) *

Primary outcomes were identified using the following OHIP fee codes: Hand/Wrist fracture repair (F004, F027, F031, F056), Cesarean-section (P018), Ureteral stent placement (E789, E817, D773, E790), Cholecystectomy (S287), Treatment of an upper tract urinary stone (E822, E760, E761, Z628), Hysterectomy (S757), Femur fracture repair (F101), Hip replacement (R440), Transurethral prostatectomy (S655), Appendectomy (S207).

^a^ Results adjusted for the binary variables of chronic obstructive pulmonary disease (COPD), cancer and heart disease.

^b^ For privacy reasons, groups of people with an n < 6 are not reported.

* p < 0.05.

Two post-hoc analyses were carried out after seeing our initial exploratory results. The first was measuring the use of nephrostomy tube insertions (OHIP code J046) during the same at-risk period as our primary outcomes (30 days after the index date until the end of follow-up). The COVID-19 cohort was significantly less likely to undergo this procedure (adjusted HR 0.251 (95% CI 0.075-0.840). The second was the frequency of requiring upper tract renal stone surgery or ureteral stent placement in the immediate 30 days after the index date; the adjusted HR for these outcomes during this earlier period were also significantly lower for the COVID-19 cohort ( Table 2).

## Discussion

In this study, we determined the relative risk of common operative procedures beyond the first 30 days of COVID-19 infection. For most of the surgical procedures, there was no significant increased risk, and many of these hazard ratios were close to one. We did however identify three surgical procedures that were significantly less likely to be carried out in patients who had a history of COVID-19 infection. To our knowledge, this has not been previously described.

A reduced risk of upper tract renal stone surgery or ureteral stent placement (HRs of approximately 0.50 for each) was observed. After seeing this result, we explored whether this could be due to an increased reliance on nephrostomy tube insertions (which generally only require sedation rather than a general anesthetic), however this procedure was rare and also significantly lower among COVID-19 patients. Similarly, the results were not explained by increased stone disease in the 30 days immediately after COVID-19 infection. Therefore, our results could be due to a reduced presentation of renal stones, a reluctance from urologists to intervene on stones after COVID-19 infection, or a lower propensity to form stones or have symptomatic renal stone disease after COVID-19. In general, the COVID-19 pandemic reduced the frequency of emergency room presentations of renal colic, and many hospitals setup triage procedures that precluded treatment of any non-urgent stone disease. ^19^ This however should have impacted both the COVID-19 and control cohort. During this time period of the pandemic, people in general were less likely to go to the emergency room with renal colic and were more likely to avoid operative intervention. ^20^ This may have been amplified in post COVID-19 patients. It is likely that non-acute renal stone interventions were avoided among patients with a recent history of COVID-19 due to uncertainties about patient and operating room personnel safety, and due to the provincial operating room procedures. ^21^ It is also possible that the specific renal effects of COVID-19 (mediated through the ACE receptors in the kidney) led to a lower propensity to develop kidney stones; the resulting kidney injury may have led to an increased focus on hydration after COVID-19 infection, patients may have experienced favorable anti-lithogenic electrolyte shifts during the acute infection, or care processes around post-COVID-19 infection follow-up may have decreased the risk of renal stone development and subsequent intervention. ^22^^,^^23^ Magnetic resonance imaging (MRI) abnormalities have also been detect in the kidney after COVID-19 infection, and these changes may impact renal stone risk. ^24^

The reduced risk of cholecystectomy was an interesting finding. Abdominal imaging in COVID-19 patients is more likely to detect biliary sludge and gallstones, ^25^ which would lead to a hypothetically increased risk of future complications and need for cholecystectomy (contrary to our findings). Similar to renal stones, it is possible that conservative management was the goal for the initial period after COVID-19, and surgical interventions were deferred for the same reasons previously discussed. ^26^

Some limitations of our study are important to acknowledge. The pandemic started in Canada in February of 2020, therefore our period of follow-up after COVID-19 infection is limited in our study to at most 10 months. Many hospitals in Ontario instituted surgical triage protocols which affected the delivery of elective care; however, our surgical procedure list does contain a mix of procedures which are traditionally elective and others that are more urgent. The time period of the study was characterized primarily by COVID-19 wildtype infections, and it is possible that the subsequent variants have different effects. People can have asymptomatic COVID-19, and early in 2020 testing for COVID-19 was limited to those who met specific criteria; therefore, some people with a prior COVID-19 infection may have been misclassified and included in the control group. The risk of this should be low, as a study of healthcare workers (a group that did have early access to testing and more stringent screening for symptoms) found that 3% had COVID-19 antibodies in the absence of a documented COVID-19 positive test. ^27^ Finally, all observational studies may have residual confounding.

## Conclusions

After a COVID-19 infection there is not an increased risk of needing common surgical procedures. There appears to be a reduced risk of renal stone disease treatment and ureteral stent placement, and a reduced risk of cholecystectomy, however understanding the reasons for this will require more study.

## Data availability

### Underlying data

The dataset from this study is held securely in coded form at ICES. While legal data sharing agreements between ICES and data providers (e.g., healthcare organizations and government) prohibit ICES from making the dataset publicly available, access may be granted to those who meet pre-specified criteria for confidential access, available at www.ices.on.ca/DAS (email: das@ices.on.ca). Further details about the process can be found here.

### Extended data

Open Science Framework: Supplementary information for Welk B, Richards L. Does CoVID-19 infection change the need for future surgical interventions? An exploratory analysis. https://doi.org/10.17605/OSF.IO/GHDB9. ^28^

This project contains the following extended data:

- STROBE/RECORD checklist
- Data collection plan
- Example analytic code

Data are available under the terms of the Creative Commons Zero “No rights reserved” data waiver (CC0 1.0 Public domain dedication).

### Analysis code availability

The full underlying analytic code are available from the authors upon request after approval for access to the data from the Data Analytic Services at ICES (see data availability statement). Users must understand that the computer programs may rely upon coding templates or macros that are unique to ICES and are therefore are either inaccessible or they may require modification.
