# Supplementary material for: Does COVID-19 infection change the need for future surgical interventions? An exploratory analysis
Source: F1000Res. 2021 Nov 17;10:1167. [Version 1] doi: 10.12688/f1000research.74861.1 (PMC8787588; doi:10.12688/f1000research.74861.1)
Supplement: Peer review history

# Peer review: Reviewer response for version 1

**DOI:** 10.5256/f1000research.78661.r115400

**Published:** 2022-01-24

**Authors:** Jorge Moreno-Palacios

**Affiliations:** 1 Mexican Institute of Social Security, Specialty Hospital, XXI Century National Medical Center, Mexico City, Mexico

The authors report regarding surgical procedures during the COVID-19 pandemic in a province of Canada. Interestingly, they found that there appears to be a reduced risk of renal stone disease treatment and ureteral stent placement, and a reduced risk of cholecystectomy. The data obtained are well presented, and although the information is new regarding the COVID-19 literature, it could not be applied to all medical systems (they acknowledge this as one of the limitations of the study), but give us the idea that due to COVID-19, surgical procedures changed according to each specific site, pandemic situation, and resources. New triage protocols for surgical procedures were developed in the hospitals and they are now performed on a regular basis. 

 Different protocols ^1^ had been suggested to ensure the surgical treatment of patients with and without the infection. Although the focus will be on treating the epidemic, patients will continue to need emergency and elective procedures so research models as the one presented in this article could help similar health systems to plan how surgical services will operate during this health crisis.

Is the work clearly and accurately presented and does it cite the current literature?

Yes

If applicable, is the statistical analysis and its interpretation appropriate?

Yes

Are all the source data underlying the results available to ensure full reproducibility?

Yes

Is the study design appropriate and is the work technically sound?

Yes

Are the conclusions drawn adequately supported by the results?

Yes

Are sufficient details of methods and analysis provided to allow replication by others?

Yes

Reviewer Expertise:

Urologist, Endourology, Functional Urology

I confirm that I have read this submission and believe that I have an appropriate level of expertise to confirm that it is of an acceptable scientific standard.

## References

[1] : Global guidance for surgical care during the COVID-19 pandemic. *Br J Surg* .107(9) : 10.1002/bjs.11646 1097-1103 10.1002/bjs.11646 32293715PMC7262310

# Peer review: Reviewer response for version 1

**DOI:** 10.5256/f1000research.78661.r100488

**Published:** 2021-12-03

**Authors:** Henry Tran

**Affiliations:** 1 Department of Urologic Sciences, University of British Columbia, Vancouver, Canada

This paper is a retrospective cohort study looking at whether COVID-19 infection increases chances of needing surgical procedures after the period of acute infection.

 The authors used the Ontario Laboratories Information System (OLIS) to determine COVID-19 PCR results, in addition to Ontario Health Insurance Plan (OHIP) records to identify all people in Ontario with a positive COVID 19 nasopharyngeal PCR test between February 1 to May 31, 2020. Control cohort was generated by identifying people who had one negative COVID nasopharyngeal PCR test between February 1-May 31, 2020. Long term care residents and patients < 18 years of age were excluded. Primary outcome was occurrence of surgical procedures 30 days after index date, identified using OHIP fee codes.

 The top 10 surgical procedures in the COVID-19 cohort were analyzed and there was no significant increased risk of needing those specific surgical procedures in the COVID-19 group. They found a reduced risk of having renal stone disease treatment and ureteral stent placement and cholecystectomy.

 The authors do a good job comparing the COVID-19 cohort with the control cohort and have a high number of patients in their analysis. They correctly identify limitations to their study, including the potential for misclassification of COVID-19 positive and negative patients due to possibility of patients with asymptomatic disease and COVID-19 testing errors.

 Overall, I feel that the paper is ready for indexing.

 This study addresses an important question of whether or not there is an increased risk of surgical diseases in COVID-19 patients.

Is the work clearly and accurately presented and does it cite the current literature?

Yes

If applicable, is the statistical analysis and its interpretation appropriate?

Yes

Are all the source data underlying the results available to ensure full reproducibility?

Yes

Is the study design appropriate and is the work technically sound?

Yes

Are the conclusions drawn adequately supported by the results?

Yes

Are sufficient details of methods and analysis provided to allow replication by others?

Yes

Reviewer Expertise:

Reconstructive Urology

I confirm that I have read this submission and believe that I have an appropriate level of expertise to confirm that it is of an acceptable scientific standard.
